# MEASURING CAREGIVING INTENSITY

**DOI:** 10.1093/geroni/igae098.3134

**Published:** 2024-12-31

**Authors:** Pamela Nadash, Eileen Tell, Sung Park

**Affiliations:** University of Massachusetts Boston, Boston, Massachusetts, United States; University of Massachusetts Boston, Boston, Massachusetts, United States; University of Massachusetts Boston, Boston, Massachusetts, United States

## Abstract

This paper discusses measurement of caregiving intensity, using empirical data to illustrate the need to develop a consensus around measurement of this construct. “Caregiving intensity” differs from the related construct, “caregiver burden”, which measures the subjective experience of caregiving; caregiving intensity focuses instead on objective measures of the caregiving experience. Caregiving intensity is operationalized in various ways, employing hours of care provided, the perceived difficulty of care, care recipient characteristics, co-residence, or combinations thereof. We use a unique dataset comprising a representative panel of 385 family caregivers, which was compiled to help understand the decision-making process behind hiring professional home care. Consequently, the survey collected rich data about the caregiving experience of participants, including caregiving-related variables and respondents’ employment situations, enabling the construction of variables representing caregiver intensity, including the frequency of care provided for activities of daily living (ADLs) and instrumental activities of daily living (IADLs) and the level of perceived difficulty in providing those activities, as well as co-residence, long-distance caregiving, and having a child under 21. Using multivariate techniques employing these measures of intensity as predictor variables, the study found that while the frequency of helping with ADLs and IADLs mattered in predicting use of paid home care, the likelihood of being employed was not predicted by frequency of ADL and IADL support, but rather by the perceived difficulty of these caregiving tasks (as well as other variables). These findings suggest the need for further work in conceptualizing caregiving intensity.

